# Biocompatibility of a Marine Collagen-Based Scaffold In Vitro and In Vivo

**DOI:** 10.3390/md18080420

**Published:** 2020-08-11

**Authors:** Dafna Benayahu, Leslie Pomeraniec, Shai Shemesh, Snir Heller, Yoav Rosenthal, Lea Rath-Wolfson, Yehuda Benayahu

**Affiliations:** 1 Department of Cell and Developmental Biology Sackler Faculty of Medicine, Tel Aviv University, Tel Aviv 69978, Israel; leslieyael.p@gmail.com; 2 Department of Orthopaedic Surgery, Rabin Medical Center, Petach Tikva, and Sackler Faculty of Medicine, Tel Aviv University, Tel Aviv 69978, Israel; shemesh.shai@gmail.com (S.S.); snirheller@gmail.com (S.H.); yoav.rosenthal@gmail.com (Y.R.); 3 Department of Pathology, Rabin Medical Center, Petach Tikva, and Sackler Faculty of Medicine, Tel Aviv University, Tel Aviv 69978, Israel; leawolfson@gmail.com; 4 School of Zoology, George S. Wise Faculty of Life Science, Tel Aviv University, Tel Aviv 69978, Israel; yehudab@tauex.tau.ac.il

**Keywords:** collagen fibers, scaffold, biomedical device, biocompatibility

## Abstract

Scaffold material is essential in providing mechanical support to tissue, allowing stem cells to improve their function in the healing and repair of trauma sites and tissue regeneration. The scaffold aids cell organization in the damaged tissue. It serves and allows bio mimicking the mechanical and biological properties of the target tissue and facilitates cell proliferation and differentiation at the regeneration site. In this study, the developed and assayed bio-composite made of unique collagen fibers and alginate hydrogel supports the function of cells around the implanted material. We used an in vivo rat model to study the scaffold effects when transplanted subcutaneously and as an augment for tendon repair. Animals’ well-being was measured by their weight and daily activity post scaffold transplantation during their recovery. At the end of the experiment, the bio-composite was histologically examined, and the surrounding tissues around the implant were evaluated for inflammation reaction and scarring tissue. In the histology, the formation of granulation tissue and fibroblasts that were part of the inclusion process of the implanted material were noted. At the transplanted sites, inflammatory cells, such as plasma cells, macrophages, and giant cells, were also observed as expected at this time point post transplantation. This study demonstrated not only the collagen-alginate device biocompatibility, with no cytotoxic effects on the analyzed rats, but also that the 3D structure enables cell migration and new blood vessel formation needed for tissue repair. Overall, the results of the current study proved for the first time that the implantable scaffold for long-term confirms the well-being of these rats and is correspondence to biocompatibility ISO standards and can be further developed for medical devices application.

## 1. Introduction

Collagen is a main extracellular matrix protein that supports the structure mainly of skeletal tissues. Collagen has a main function during the healing process and in cases when the tissue will not heal spontaneously, there is a need to foster it. These bring the collagen to serve both as an essential protein and a major component in biomedical scaffolding for various tissue regeneration approaches. The scaffold facilitates and promotes the autologous stem cells growth and differentiation that will progressively enter the scaffold and replace it by the regenerating tissue. The tissue-engineered scaffolds approach mimics the natural tissue structure and physical properties of the targeted tissue. Such an approach also aims to minimize the use of autologous grafts, which are limited by the availability of the patient’s own tissue and may avoid additional surgical procedures. Thus, the challenge is to find a suitable source of collagen, whose extraction and purification procedures will be suitable for medical applications. The overall goal is to find a replacement to synthetic polymer materials used as medical devices, which do not integrate with the body and may trigger an immune response such as chronic inflammation [1]. The use of natural materials as scaffolds is beneficial, and recently it has been shown that certain marine organisms are a promising bio-source to obtain collagen for scaffold formation in a variety of biomedical applications. The great interest in the field is highlighted by a series of studies for biomaterial isolated from different marine species allow assorted applications as biomedical devices [2,3,4,5,6,7,8,9,10,11].

Collagens are a heterogeneous family of extracellular matrix proteins, but are highly conserved through evolution. The collagen structure is highly homologous among invertebrates and mammalians, including mice and humans, which allow the collagen to be metabolically compatible through different phylogenetic groups. The collagen family is divided into fibrillar and non-fibrillar categories based on packing and ultrastructure, and this study focus on a fibrillar collagen that was studied in detail and described by us for its molecular and ultrastructure [12]. Marine sources with a high quantity of collagen are bio-compatible with mammalian cells in vitro [13]. The natural materials are biological macromolecules, which are considered to minimize immunological reactions [14,15,16]. Another advantage of using marine-derived collagen over the mammalian one is that invertebrates can be cultivated [17] under controlled conditions for the purpose of collagen extraction, thus having regulatory and quality control allowing overcoming ethical constraints for use in medical applications.

Most invertebrate collagens appear as extracellular matrices, and their mechanical properties are limited. In this study, the unique collagen fibers were shown to be extracted by a rather simple procedure by pulling out from the coral tissues [6,13]. These unique coral-derived collagen fibers were identified for their mechanical properties [6,18,19,20], which attribute the bio composite suitability for the formation of scaffolds that can be tailored to meet the mechanical properties of the target tissues. The bio composite developed in this study used collagen fibers which were embedded in alginate hydrogel [6,13], allowing the formation of a three-dimensional scaffold that supports cells growth, as was recently studied for its biocompatibility in vitro, and proved to support cell growth and differentiation [13]. The collagen fibers in the bio-composite provide mechanical and biological cues for cell proliferation and tissue regeneration. An additional molecular cue can be added into the bio composite using nanoparticles (NPs) as a delivery of growth factors, providing biological cues that modulate and promote cell proliferation and differentiation into a desired lineage fate. Based on in vitro studies, the efficiency of cells to endocytose the NPs that were continued to proliferate and differentiate [13,21].

This study further analyses the biocompatibility of the bio composite in vivo in a rat animal model. The experiment demonstrated the healing of a rotator cuff tear, the most common musculoskeletal injury occurring in the shoulder [22]. The bio composite implant served as an augment facilitating tendon repair by relief the load from the healing tendon and eventually allowing both the restoration of this mechanosensitive tissue and the mobility of the operated extremity. In addition, it also served separately as a subcutaneous implant. Following the biocomposite implantation in two sites, the rats were followed up for any sign of the material cytotoxic effects and followed up on their viability, well-being, and functionality. For any new material such as the bio composite presented here, the follow-up is required for biomedical development and resulted in no indication of any toxicity of the material. Thus, the material tested in vivo for biocompatibility to meet the standards established by the Food and Drug Administration Organization for the sub-chronic toxicity (ISO-10993) of transplants, and improved the physiology of the operated rats. The designed biomaterial will allow the future development of bio composite-based products with optimal mechanical properties that will fully integrate with the natural tissue, contributing to its healing processes.

## 2. Results

**Figure 1:**
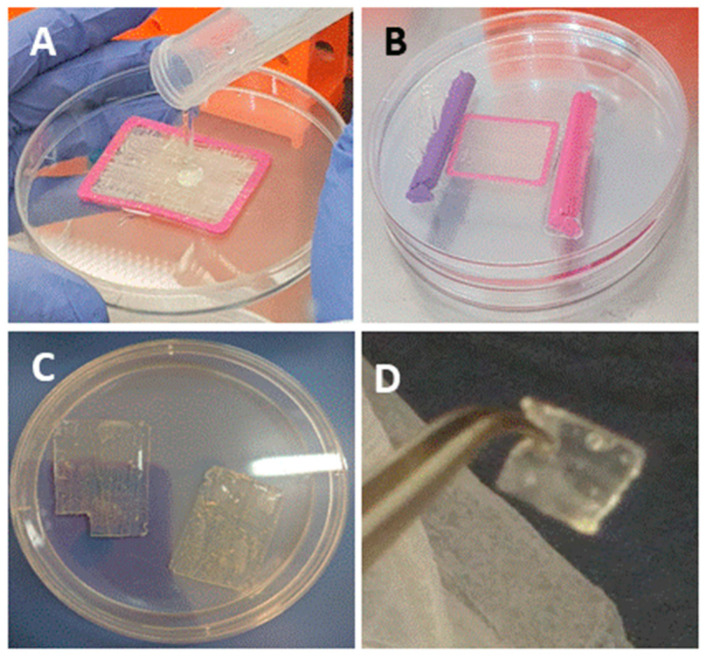
Bio composite preparation. (**A**) Collagen fibers spun around a PLA frame embedded in alginate solution; (**B**) the frame with collagen fibers and alginate inserted in a dialysis bag and immerse in calcium solution to allow alginate gelation; (**C**) the resulting bio-composite device was stored in ethanol solution and washed buffer before use; (**D**) collagen-alginate hydrogel device used for supraspinatus tendon augmentation.

**Figure 2:**
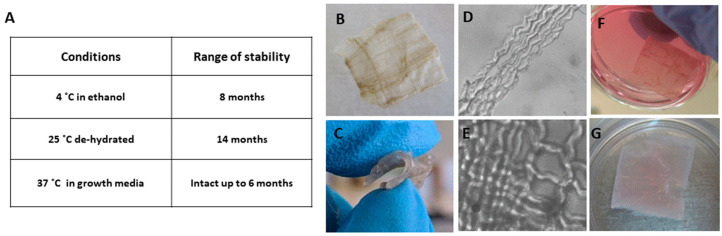
(**A**) Table summarizing the conditions that bio composite films were stored in: storage durations dry or wet material when immersed in 70% ethanol or in growth medium for long periods (at least 6–14 months), at temperatures (4 °C, 25 °C and 37 °C), as indicated in each experiment. (**B**–**G**) Pictures show the bio composite under various conditions, (**B**) biocomposite dry material, (**C**) rehydrated samples from B in PBS, showing its elasticity. (**D**) and (**E**) magnification of the collagen fibers preserving their structure in the biocomposite. (**F**) Bio-composite film kept at 37 °C in cell growth medium. (**G**) Bio-composite film kept at 4 °C in ethanol.

**Figure 3:**
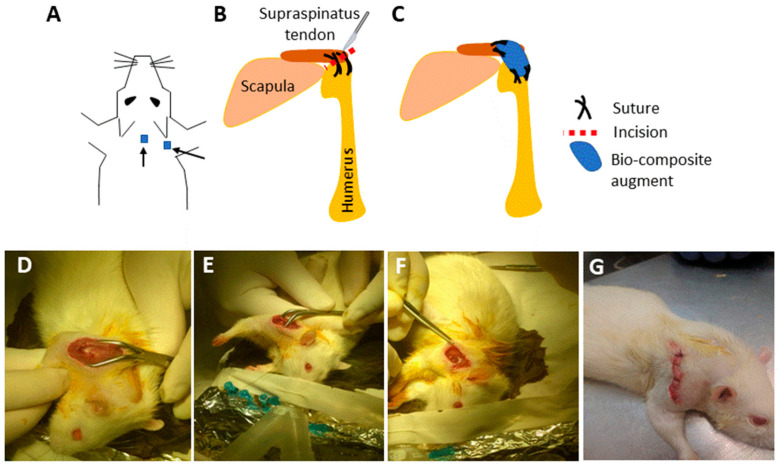
Surgical procedure. For rotator cuff tearing, repair, and augmentation with collagen-alginate film. (**A**) Implantation sites for subcutaneous and augment implants; (**B**) surgical tearing and repair of the supraspinatus; (**C**) augmentation with bio-composite film; (**D**) exposure of the supraspinatus tendon. (**E**) Suturing the tendon before cutting; (**F**) augmentation of supraspinatus tendon; (**G**) rat after surgical tearing, repair, and augmentation of the rotator cuff tendon.

The bio-composite production is demonstrated in Figure 1 and a detailed procedure is described in the Materials and Methods section. We analyzed the bio-composite for stability under various storage conditions: (I) dry film was air-dry at room temperature for 14 months, (II) film stored at 4 °C immersed in 70% ethanol for 8 months, (III) film was stored at 37 °C in cell growth media for 6 months. The follow up of dry bio-composite film after 14 months presented no signs of powdering or tearing of the alginate film. The film was immersed in buffer for re-hydration, and the material demonstrated a good stability and the recovery of elasticity, along with the preservation of structure and the organization of the collagen fibers embedded in the alginate. Thus, the dry re-hydrated material was fully functional as a scaffold (Figure 2A–C). Similarly, the composite structure was preserved when the film was maintained at 4 °C in 70% ethanol for 8 months (Figure 3A,D,E). As for the bio composite film immersed in cell growth media at 37 °C for 6 months, visual inspection revealed that the alginate displayed signs of disintegration on its surface, whereas the collagen fibers strengthened the bio-composite and maintained the structure (Figure 2A,F,G). These results confirm the in vitro stability of the bio-composite even when stored for long periods under different conditions, as dry or wet material.

**Figure 4:**
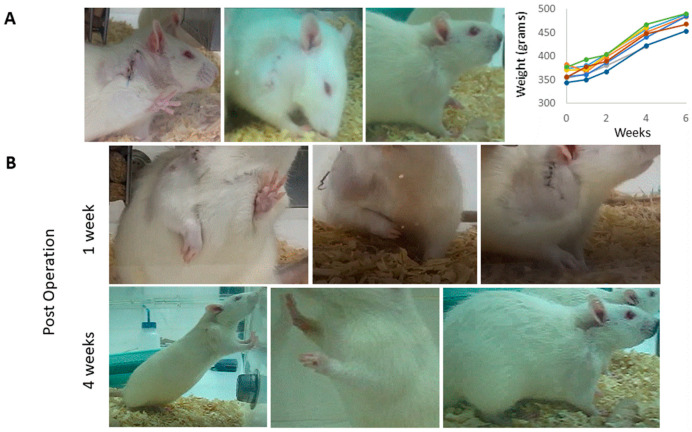
Animal recovery after surgery. (**A**) Recovery follow up of the operated site wound healing and animal weight; (**B**) physical signs for right rotator cuff function impairment 1 week after surgery (middle raw), bending and keeping the right front limb close to the chest, avoid leaning on the operated limb and tendency to step or stand on the left foreleg and hind limbs/short step on the operated foreleg and fast weight shifting to the left foreleg. After 4 weeks, the operated site displayed complete healing, and rats’ motility recovery allows them to perform daily activities.

The bio composite film aimed to serve as scaffold for tissue repair was recently used in a cell culture system and analyzed for growth, migration, and differentiation in 2D and 3D scaffolds in vitro [13]. In the current study, we applied the bio composite film as medical device for tissue repair in vivo at two sites in rat. In one site, the scaffold was used as an augment for aid of repair of a torn rotator cuff supraspinatus tendon, and the other was transplanted in sub-cutaneous site. For tendon repair, a unilateral surgical detachment of the rotator cuff supraspinatus tendon model was utilized. The tendon detachment was then sutured and an augment of bio-composite collagen-alginate was laid on the repair site. The purpose of the transplantation of the augment was in order to contribute to the tendon recovery by relieving the overall load during the healing process. The rat mobility was monitored by video during the rotator cuff recovery period (Figure 3 and Figure 4). It was observed immediately after surgery that the rat lost the proper function of the operated extremity. The rat had impaired motion and avoided leaning on the operated foreleg, while bending and keeping the right front limb close to the chest (Figure 4 and Video S1). The recovery follows up of operated rats included monitoring of animal weight and demonstrated the animal wellbeing along with the weight rising over the weeks (Figure 4). In addition, the Rats’ daily follow up in order to confirm their well been there was no signs of depression or suffering. Follow up by physical examination demonstrated healing of the external surgical wound, together with animals’ recovery of their mobility (Figure 4B). At first after operation, the rats demonstrated the tendency to step on the left foreleg and hind legs, while bending and keeping the right front limb close to the chest (Figure 4 and Video S1). As time passed, the operated rats displayed comfort stepping on the recovered limb (Video S1) and the healing of the external surgical wound (Figure 4). Altogether, the results demonstrated no adverse responses or any sign of cytotoxicity to the transplant in the operated animals.

**Figure 5:**
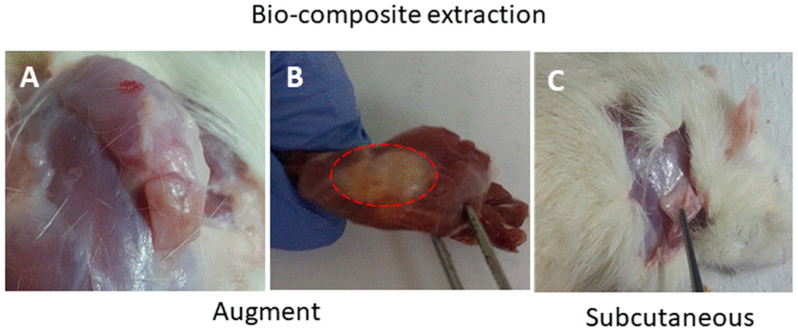
Macro observation of the bio composite film transplants extracted from the augment over rotor cuff (**A**–**C**). Sub-cutaneous site.

**Figure 6:**
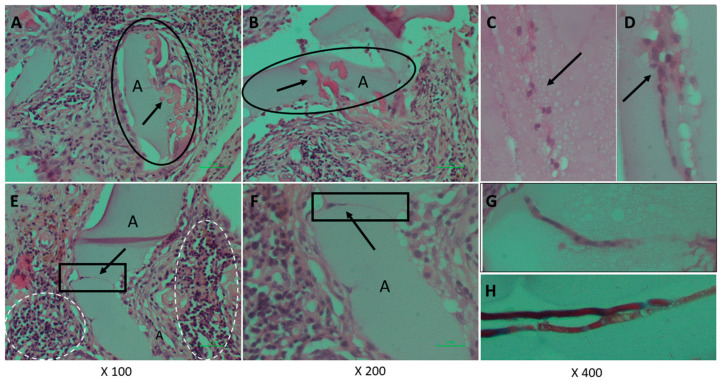
Histopathological examination of the bio-composite transplant retrieved from subcutaneous stained by haematoxylin and eosin shows the alginate as an amorphic material (light pink, in elliptic circle) marked in **A** and the collagen fibers embedded (darker pink, marked by arrow). Around the bio-composite, a fibrous tissue is observed and clusters with lymphocyte and plasma cells infiltration (nuclei stained in blue, marked in a white dashed circle). At (**C**,**D**,**G**,**H**) (arrow or square), a blood vessel that is developing through the alginate material is seen. Magnifications are (**A**,**E**) × 100; (**B**,**F**) × 200; (**C**,**D**,**G**,**H**) × 400.

**Figure 7:**
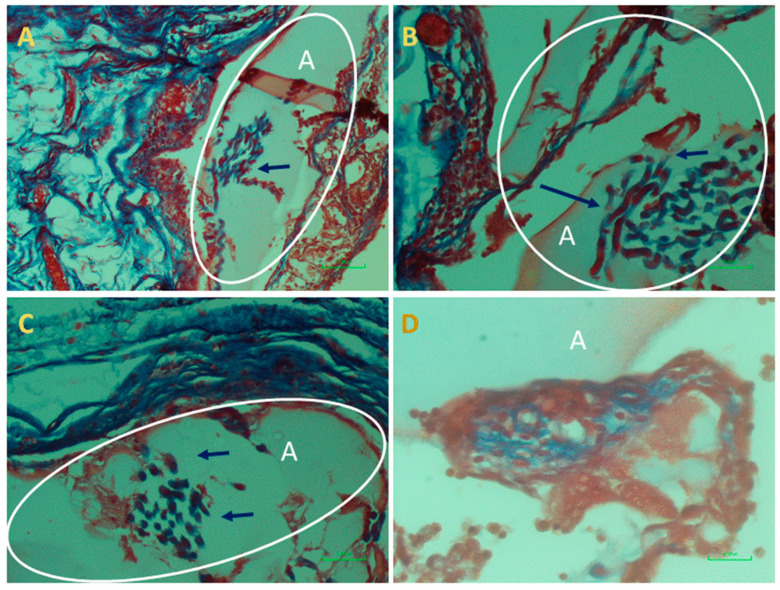
Histopathological examination of the augment bio-composite transplant stained by Masson trichrome seen in (**A**–**D**). Alginate is an am orphic material (light pink, marked in **A** and the collagen fibers embedded in the alginate (marked by arrow, darker blue). Around the bio-composite, a fibrous tissue (blue) is observed.

At the end of the experiment, the scaffolds transplanted as augments or at subcutaneous sites and the surrounded tissues were extracted and examined histologically. This analysis was performed in order to assess cells’ interaction with the bio-composite and a potential cytotoxic effect or foreign body response. The results visualized that the transplanted bio-composites were integrated with the surrounding tissues, as seen at the macro-level as a square cube surrounded by a fibrotic tissue growing over (Figure 5). The histological analysis revealed that the tissue was formed around and inside the scaffold, (the alginate is visible as am orphic material and marked by A in Figure 6 and Figure 7, and the collagen fibers are marked by arrows). Around the bio composite, the following findings were noted: inflammatory cell recruitment and differentiation to lymphocyte and plasma cells and macrophage activation fused to form giant cells. These findings are expected to be a response to foreign material associated with formation granulation tissue and fibroblast being part of the inclusion process of this material (Figure 6 and Figure 7). The formation of new blood vessels developing through the alginate material was noted (Figure 6). Masson trichrome staining demonstrate that the fibrous tissue found around the bio-composite developed inside the scaffold along (Figure 7).

## 3. Discussion

Implant integration with the healing tendon relies on autologous cells function during tissue regeneration, and the augment facilitated the improvement of this recovery step. In the current study, we aimed to evaluate the stability of the bio-composite augment. We analyzed the produced bio composite film both in vitro [13] and in vivo to examine the material stability and its biocompatibility, cytotoxicity, and potential for tissue repair.

First, we evaluated in vitro the stability of the collagen-alginate hydrogel bio composition device under different storage conditions (Figure 2). The bio-composite maintained the natural structure of collagen fibers was stable for at least 14 months as dry material with no sign of film powdering. When the film was hydrated in phosphate buffer to recover its elasticity, no effect on the structure and organization was noted. In addition, the film stored in 70% ethanol at 4 °C displayed the conservation of the bio composite integrity. When the bio-composite film was incubated at 37 °C in growth medium, only superficial hydrogel was disrupted, while the supporting fiber structure in the film and its’ shape were kept intact. Thus, the device is stable for long periods (at least 6–14 months) as dry and wet material at a wide range of temperatures (4 °C, 25 °C and 37 °C).

A biocompatibility study recently showed the properties of the bio-composite for cells growth and differentiation in vitro with no sign of cytotoxicity for period of up to 11 weeks [13]. The current study, aimed to follow the scaffold in-vivo where the collagen-alginate scaffold film was transplanted in rats at two locations: (1) subcutaneous and (2) for rotator cuff tendon tearing as an augment. The two experimental sites allowed the biofilm to contact with different tissues. At the subcutaneous site, it was next to the hypodermis of the skin, while the augment was next to the tendon and muscle, a rich vascularized tissue. When the devices were extracted from the operated animals, they were found at the transplanted sites and seen intact. These results indicate that the bio-composite film display stability in vivo up to 14 weeks and proved to be a successful scaffold during the healing process.

The rats were analyzed for their recovery, and the animals displayed the disruption of the left rotator cuff function, as shown in Figure 4. The rats’ tendency was to protect the operated limb, bending it close to the chest, and avoiding standing and stepping in this foreleg. During recovery follow up, the operated rats recovered and were back to normal use of the operated limb. After four weeks, all the animals displayed normal mobility with no difference between right (operated) and left rotator cuff and no preference for one of them for activity. This result indicates that the presence of the scaffold augment protects the tendon and tissue regeneration during the healing processes. In these experiments, the rats were with good appetite, increased their weight, were back to normal activity and social non-aggressive behavior, with no signs of stress or suffering along the study, and up to complete curing (Figure 4)—i.e., no sign of cytotoxicity on these rats. Rats were sacrificed and the scaffolds were taken for a histology analysis to evaluate the transplant biocompatibility. The transplants were found to be integrating with the surrounding tissues and encapsulated in the granulation tissue, which is a stage of the healing process and a normal and expected response. A mild immunological response was observed by presence of plasma cells and macrophages, known to secrete pro-fibrogenic factors, which enhance fibro genesis, and is common at this stage. Thus, a fibrous capsule that developed around a transplanted material inhibit macrophages’ activity by avoiding their reaching the transplant. Therefore, it is concluded that during the experimental time, the bio-composite device has no negative effect on animals’ health, and that this in vivo assay matched the in vitro previous cytotoxicity research [13].

The mechanisms underlying tendon healing encompass the step of macrophages recruitment, followed by fibrous tissue formation and the regulation of ECM remodeling. The presence of fibro-proliferation is proven and angiogenesis lead to tissue remodeling during the wound’s healing. Such steps were observed during the current in vivo rotor cuff tendon augmentation and subcutaneous transplant. These findings emphasize the biocompatibility of the bio-composite and its safety, which is a principal quality for clinical use. Therefore, it is concluded that the analyzed material meets the ISO standard for biocompatibility.

## 4. Material and Methods

### 4.1. Bio-Composite Preparation and In Vitro Storage and Stability Analysis

#### 4.1.1. Collagen Fiber Isolation

Coral collagen fibers were isolated by mechanical extraction from the soft coral *Sarchophyton ehrenbergi* [6,13,17]. The isolated fibers were manually spun around a thin polylactic acid (PLA) frame to create a dense net of multidirectional fiber bundles. The extracted fibers were washed thoroughly in several solutions—distilled water, 0.1% sodium dodecyl sulphate, 0.5 M ethyl enediamine tetraacetic acid (EDTA), and phosphate buffered saline (PBS)—and immersed in ethanol 70% until bio-composite fabrication.

#### 4.1.2. Alginate/Collagen Bio-Composite Fabrication

Next, 3% (*w*/*v*) alginate solution was produced by dissolving sodium alginate (Protanal LF 10/60, FMC BioPolymer, Philadelphia, PA, USA) in distilled water. The isolated collagen fibers were arranged in various orientations on a frame to provide the mechanical properties to the biocomposite. The collagen fibers were embedded in alginate and then inserted into a dialysis membrane (6000–8000 MWCO, Spectra Por, Spectrum Labs Inc., Rancho Dominguez, CA, USA). The membrane was sealed, flattened, and soaked for 24 h in a calcium-containing solution at physiological concentration (0.02 M CaCl_2_). Calcium divalent cations mediate cross-linking between the polysaccharide chains of the alginate, which become a hydrogel (Figure 1). The bio-composite was removed from the dialyzed membrane and the frame, and then stored in 70% ethanol at 4 °C. Before implantation, the bio-composite transplants were washed in phosphate buffered saline (PBS).

#### 4.1.3. Bio-Composite In Vitro Storage and Stability

The stability of the bio-composite film was analyzed under various conditions—dry or immersed in 70% ethanol, and in growth medium for long periods (at least 6–14 months) at a wide range of temperatures (4 °C, 25 °C, and 37 °C) as indicated in each experiment.

### 4.2. Rat In Vivo Study

Bio composites of collagen/alginate were transplanted in Wister male rats 12-weeks old (average weight of 350 gr) with the approval of Tel Aviv University Institutional Animal Care and Use Committee Number 01-18076. The rats underwent a unilateral detachment of the right supraspinatus tendon, and were subjected to repair and augmentation with a bio composite scaffold implant as described in the following surgical procedure of rotator cuff augmentation or sub-cutaneous implantation sites. Rats were anesthetized with 5% isoflurane inhalation (maintained at 2–3% during surgery) and injected with Rimadyl (NSAID) prior to surgery. All the animals were subjected to identical unilateral supraspinatus detachment and repair. The animals were operated on the right shoulder, where a 1.5 cm skin incision was performed over the anterolateral border of the glen humeral joint. The deltoid was exposed and the supraspinatus tendon was identified (Figure 3). The supraspinatus tendon was then cut to its’ full thickness, with a blade (No 15) just proximal to the tendon-bone insertion at the greater tubercle (rotator cuff footprint). A horizontal mattress suture was placed; passing horizontally through both sides of the detached tendon and tied over the proximal side using Vicryl 2-0 (Ethicon, Somerville, NJ, USA). Subsequently, an augmentation bio composite film of 5 × 5 mm^2^ was placed over the repaired supraspinatus tendon and sutured down to the supraspinatus with Vicryl 4-0 (Ethicon, Somerville, NJ, USA). Finally, an additional subcutaneous implantation was performed, using a similar bio composite film scaffold that was placed under the skin at the back of the neck. The wound was closed in layers: the subcutaneous layer was closed with Vicryl 3-0 (Ethicon, Somerville, NJ, USA) and the skin was sutured with Vicryl 3-0 (Ethicon, Somerville, NJ, USA). The operated rats were followed up by observation until awakening from anaesthesia and daily monitored after surgery for well-being and physiological recovery. The follow up was carried out by the visual monitoring of the rats’ activity (see Video S1 and Figure 4) and weighing. During the experiment, the animals were permitted unrestricted cage activity. The rats were kept in a conventional facility with 12 h light/dark cycles and were fed with standard chow and provided water ad libitum.

### 4.3. Histology

The rats were sacrificed and the transplanted bio-composite films, which served as tendon augment or for subcutaneous implantation, were extracted and processed for histology. These analysed transplants were removed and fixed in 4% paraformaldehyde, embedded in paraffin and 5 µm thick sections were stained with haematoxylin eosin (HE) or Masson Trichrome (MT) to highlight the fibrous tissue. The slides were observed and photographed on a light microscope (Nikon, Tokyo, Japan).

## 5. Conclusions

The biocompatibility of the collagen-alginate bio composite film was previously assessed in vitro, showing that the scaffold meets the terms of the IS0 10993-5 standards for cytotoxicity. In the in vitro study, mesenchymal stem cells succeeded in migrating and colonizing the scaffold, grew and proliferated, and formed tissue-like structures along and between the collagen fibers [13]. In the current study, the material was transplanted in vivo as scaffolds in two anatomical sites, exhibiting no toxicity for evaluated by the well-being of the rats (increasing their body weight and activity after the surgery for period of few weeks). The surrounding tissues around the implant were evaluated for inflammation reaction and scarring tissue formation, which indicate the body reaction towards the implant. This study demonstrated the collagen-alginate device’s biocompatibility integration with the rats’ tissue and well physiology. The scaffold allowed the formation of a 3D structure, enabling cell migration and new blood vessel formation, needed for tissue repair. These results showing the overall well-being of the rats during the in vivo experiment follow up for a period of 14 weeks. The study proved the necessary boundary of biocompatibility of the scaffold as an implantable long-term medical device, corresponding to the ISO standards for implantation and sub-chronic toxicity (ISO 10993-6 and ISO 10993-11). This is essential step that will allow us to further develop the bio composite for various applications and compare the benefits of the new scaffold to other available ones.

## Supplementary Materials

The following are available online at https://www.mdpi.com/1660-3397/18/8/420/s1, Video S1: Follow up of animal recovery.
